# Exploration of Small Non-Coding RNAs as Molecular Markers of Ram Sperm Fertility †

**DOI:** 10.3390/ijms26062690

**Published:** 2025-03-17

**Authors:** Mustafa Bodu, Mustafa Hitit, Huseyin Donmez, Abdullah Kaya, Muhammet Rasit Ugur, Erdoğan Memili

**Affiliations:** 1 College of Agriculture, Food and Natural Resources, Cooperative Agricultural Research Center, Prairie View A&M University, Prairie View, TX 77445, USA; mbodu@selcuk.edu.tr; 2 Department of Reproduction and Artificial Insemination, Faculty of Veterinary Medicine, Selcuk University, Konya 42005, Türkiye; 3 Department of Animal Genetic, Faculty of Veterinary Medicine, Kastamonu University, Kastamonu 37100, Türkiye; 4 Division of Pharmacy and Optometry, Faculty of Biology Medicine and Health, University of Manchester, Manchester M13 9PL, UK; dr.huseyin.donmez@gmail.com; 5 Department of Animal and Dairy Sciences, College of Agricultural and Life Sciences, University of Wisconsin–Madison, Madison, WI 53558, USA; abkaya2@gmail.com; 6 IVF Michigan Fertility Centers, 37000 Woodward Ave #350, Bloomfield Hills, MI 48304, USA; mrasit20@gmail.com

**Keywords:** ram, sperm, fertility, small non-coding RNAs, miRNA

## Abstract

The identification of molecular markers for fertility is critical for the sustainability of livestock production. We profiled small non-coding RNAs (sncRNAs) in sperm from rams with high fertility (HF) and low fertility (LF) phenotypes to uncover their roles in ram sperm fertility. Rams were categorized into high-fertility (HF, *n* = 31; 94.5 ± 2.8%) and low-fertility (LF, *n* = 25; 83.1 ± 5.73%) phenotypes based on pregnancy rates (average 89.4 ± 7.2%). From these, sperm samples of HF (*n* = 4; pregnancy rate 99.2 ± 1.6%) and LF (*n* = 4; pregnancy rate 73.6 ± 4.4%) rams underwent sncRNA sequencing. Small RNA sequencing produced 14,962,876 reads in LF rams and 17,401,094 reads in HF rams, showing distinct sncRNA biotypes, including miRNAs, tRNAs, snoRNAs, snRNAs, and rRNAs. Among these, miRNAs comprised 7.12% of reads in LF rams and 3.78% in HF rams, while rRNAs and repeats formed significant proportions in both groups. A total of 1673 known and 627 novel miRNAs were identified, with 227 differentially expressed miRNAs between the HF and LF groups. We showed that key miRNAs, such as oar-miR-200b and oar-miR-370-3p, were upregulated in HF sperm, while downregulated miRNAs in LF, such as oar-miR-26b and oar-let-7d, were associated with impaired sperm function and DNA fragmentation. A functional enrichment analysis of miRNA target genes highlighted pathways related to ribonucleoprotein complex biogenesis, RNA processing, and gene expression regulation. These findings establish the critical role of sperm sncRNAs as regulators of fertility and potential biomarkers in breeding soundness tests for the precision farming of livestock for global food security.

## 1. Introduction

Ram fertility, the ability of a male to successfully fertilize an egg with a viable sperm, is essential for animal reproduction. This process ensures the continued existence of the species through embryonic and fetal development. Since rams make up over half of the genetic material in every sheep farmer’s flock, fertility is a highly valuable attribute from an economic perspective [1]. Therefore, the efficacy and productivity of sheep farming depend on the correct management of rams, so they maintain maximum performance and longevity [2]. Hence, it is crucial to enhance the fertility of male sheep in the livestock industry in order to meet the growing worldwide demand for animal-based food and sustain the needs of the expanding human population.

Alongside genetic evaluation and testing, rams have been carefully chosen based on breeding soundness evaluation (BSE). This comprehensive assessment involves a range of examinations, including a physical exam, scrotal measurement, and an analysis of sperm morphology and motility. Despite the BSE method being commonly used to evaluate ram fertility, most idiopathic infertility cases are not able to be identified using this method. A number of molecular determinants regulating sperm fertility, such as DNA damage, RNA molecules [3], and protein markers [4,5], cannot be detected using such traditional methods [6]. More advanced omics approaches have led to significant advancements in understanding the molecular mechanisms involved in spermatogenesis, fertilization, and embryogenesis [7,8,9]. These techniques can become more widely utilized in the future for the production of farm animals. They can be utilized in combination with the evaluation of semen parameters and the estimation of accurate sperm fertility markers in farm animals.

Along with the paternal DNA, sperm have a diverse content of RNAs with different nucleotide lengths and biogenesis, in addition to mRNAs, including small non-coding RNAs, such as PIWI-interacting RNAs (piRNA), microRNAs (miRNA), endogenous small interfering RNAs (endo-siRNA), and tRNA-derived small RNAs [10,11,12]. These RNA molecules are required for precise transcriptional and post-transcriptional regulation of spermatogenesis [13,14]. They play vital functional roles in the fertilization process through the delivery of low abundances of miRNA and piRNA to the oocyte [4,15]. Moreover, paternal origin sncRNAs are essential for preimplantation embryos [16] as they help maintain gene expression in the early stage of development upon fertilization [17]. Therefore, sperm sncRNAs can contribute to the transportation of epigenetic information throughout the offspring [18].

Recently, with the advance of RNA sequencing technologies and reliable bioinformatic information, sperm-born RNA was identified [19] and reported to be conversed among several species [20]. However, the comprehensive profiling of fertility-associated sncRNAs in sperm revealed that their identities and functions are largely unknown. Therefore, revealing the relationship between sncRNA and fertility will expand our understanding of the underlying biological system which determines ram fertility. The objective of this study is to profile the small non-coding RNAs (sncRNAs) in sperm from rams with HF and LF phenotypes to uncover their roles in ram sperm fertility. Such new knowledge will also aid in providing accurate insights into the selection of ram in an integrated approach, considering their genetic background and sncRNA expression to predict fertility.

## 2. Results

### 2.1. Data Quality Summary

The sequencing quality for the LF and HF sperm samples was assessed to ensure data reliability. The LF sample generated 14,962,876 reads with a total base count of 0.75G, while the HF sample produced 17,401,094 reads with 0.87G bases. Both samples showed a low error rate of 0.01%, indicating high sequencing accuracy. A quality score analysis revealed that 98.98% of bases in the LF sample and 98.52% in the HF sample had a higher Q20 score, with a Q30 score of 96.62% for the LF sample and 95.45% for the HF sample. In addition, the GC content was consistent between samples, measuring 53.11% in the LF sample and 52.99% in the HF sample (Table 1).

The LF and HF samples were evaluated for read and base compositions to assess uniqueness and overall coverage. The LF sample contained 9,851,512 total reads, corresponding to 269,292,004 bases, while the HF sample had 10,611,165 total reads, resulting in 303,347,912 bases. Unique read counts were relatively close, with the LF sample having 1,550,984 unique reads and the HF sample containing 1,494,593 unique reads. Similarly, the total numbers of unique bases were 40,934,899 for the LF sample and 41,404,805 for the HF sample (Table 2). Clean processed data are provided in the Supplementary Material (Supplementary File S1).

### 2.2. Length Distribution

**Figure 1:**
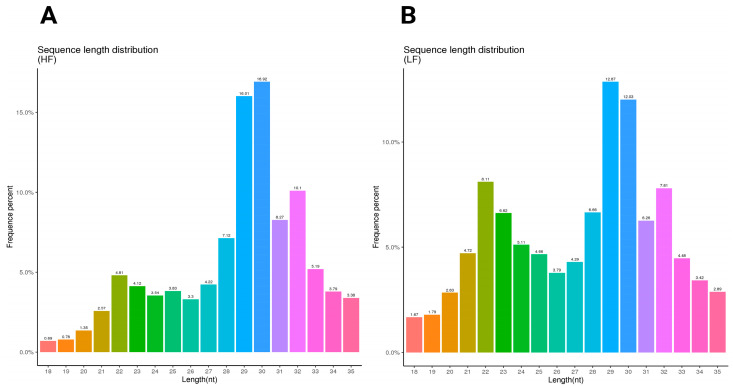
The sequence length distribution of the HF and LF samples. The length distribution and frequency percentage of small non-coding RNA sequences in ram sperm. (**A**) The sequence length distribution of the high-fertility (HF) sample; (**B**) the sequence length distribution of the low-fertility (LF) sample. The HF graph exhibits a more pronounced peak at 29 nt (16.92%), after which it declines noticeably. In contrast, the LF graph displays a broader peak spanning 29–31 nt, with a maximum frequency of 12.87% and a more gradual decline. Furthermore, the LF condition shows a higher occurrence of shorter sequences (22–24 nt) compared to the HF sample.

The sequence length distribution for the HF and LF samples shows distinct patterns in nucleotide length and frequency. In the HF sample, the most frequent sequence length is 30 nucleotides, comprising 16.92% of the total, followed closely by 29 nucleotides at 16.01%. Other notable peaks included 31 nucleotides at 8.27% and 32 nucleotides at 10.1%. This distribution indicates a concentration around the 29–32 nucleotide range (Figure 1A). In contrast, the LF sample has the highest frequency at 29 nucleotides (12.87%), followed by 30 nucleotides at 12.03%. Lengths of 22 and 32 nucleotides were also relatively frequent at 8.11% and 7.81%, respectively (Figure 1B) (Supplementary File S2).

### 2.3. sncRNA Analysis and Annotation

**Figure 2:**
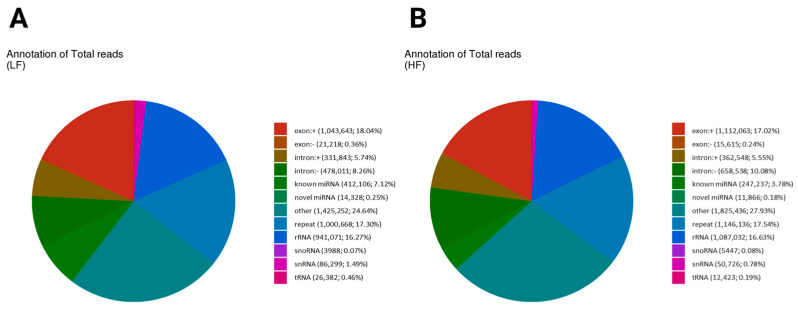
The distribution of total reads of non-coding RNA biotypes. Pie charts depicting the distribution of total reads of different biotypes of RNA species present in the sperm sncRNA datasets. The number and percentage of sRNAs reads mapped to exon (+/−) and intron (+/−)/known and novel miRNA/RNA/tRNA/snRNA/snoRNA/repeat. Different colors denote the types of RNA species. Both graphs indicate that the “other” category is the largest, with slightly higher values in the HF sample (27.93%) than the LF sample (24.64%). Repeat elements are similar in both (LF: 17.3%, HF: 17.54%). Exonic reads are slightly higher in the LF sample (18.04%) than the HF sample (17.02%), while intronic reads are more prevalent in the HF sample (intronic+: 5.55%, intronic−: 10.08%) compared to the LF sample (intronic+: 5.74%, intronic−: 8.26%), suggesting differences in transcript processing. (**A**) The annotation of total reads in the low-fertility sample (LF); (**B**) the annotation of total reads in the high-fertility sample (HF).

Small RNA reads were annotated with sequences from the non-coding transcript sequences of the *Ovis aries* with reference (Supplementary Figure S1). We show the annotation breakdown of total sncRNA reads in the LF and HF samples in the two pie charts, highlighting differences in the proportions of various RNA categories. In the LF sample, the most prominent category was the other, comprising 24.64% (1,425,252) of the total reads, indicating that a significant portion of the reads did not fit into the known RNA categories, similarly to the HF sample with 27.93% (1,825,436) (Figure 2A,B). Known miRNA reads form a substantial portion in both groups, with the LF sample having a value of 7.12% (412,106) and that of the HF sample being lower at 3.78% (247,237). Ribosomal RNA (rRNA) also contributed significantly, representing 16.27% (941,071) in the LF sample and 16.63% (1,087,032) in the HF sample. Repeats constituted 17.3% (1,000,668) in the LF sample and 17.54% (1,146,136) in the HF sample. The exon-related reads were slightly higher in the LF sample (18.40%) compared to the HF sample (17.26%), while intronic reads also showed a slight difference, with the LF sample at 13.92% and the HF sample at 15.63%. Small nucleolar RNAs (snoRNA) and small nuclear RNAs (snRNA) represented minor proportions in both groups, with each comprising less than 1% of the total reads. Small nucleolar RNA (snoRNA) was more frequent in the HF sample (0.08%) (5447 reads) than in the LF sample (0.07%) (3988 reads). Small nuclear RNA (snRNA) was also more abundant in the LF sample (1.49%), with 86,299 reads compared to the HF sample’s (0.78%) 50,726 reads. Transfer RNA (tRNA) showed a higher total in the LF sample at 0.46% (26,382 reads) compared to the HF sample at 0.19% (12,423 reads) (Figure 2A,B). The total unique breakdown of total sncRNA reads in the LF and HF samples is shown in Supplementary Figure S2.

### 2.4. Analysis of Known miRNA

#### 2.4.1. miRNA First Nucleotide Bias Across Length Variants in HF and LF Samples

**Figure 3:**
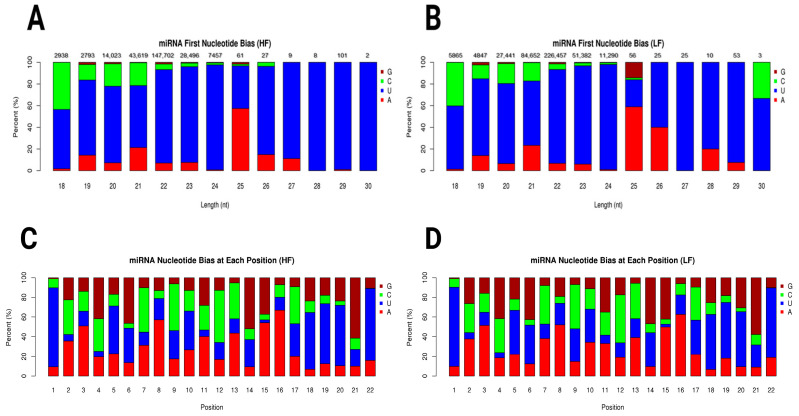
Graphs show the miRNA length distribution and nucleotide bias for HF and LF conditions. (**A**) miRNA first nucleotide bias in high-fertility ram. (**B**) miRNA first nucleotide bias in low-fertility ram. Both conditions peak at 22 nt, with LF sample having higher abundance (226,457 counts; LF) compared to HF sample (147,702 counts; HF). Distribution declines sharply for lengths shorter or longer than 22 nt. (**C**) miRNA nucleotide bias at each position in high-fertility ram. (**D**) miRNA nucleotide bias at each position in low-fertility ram. Nucleotide bias graphs suggest position-specific preferences, crucial for miRNA function, such as uracil (U) preference at 5′ end. Uracil (U), Adenine (A), Guanine (G), and Cytosine (C).

The miRNA first nucleotide bias plots for the HF and LF samples showed distinct patterns of nucleotide preferences across different miRNA lengths. In the HF sample, uracil (U) predominated across most miRNA lengths, especially in the 22–24 range compared to the 26–30 nucleotide range. Adenine (A) showed a modest presence, particularly in miRNAs of 25 nucleotides in length. Guanine (G) and Cytosine (C) were less represented in shorter miRNAs but appeared in notable quantities in specific lengths (Figure 3A). In the LF sample, a similar preference for uracil was observed, although the proportion of Cytosine (C) was slightly more pronounced in some length categories, particularly in shorter miRNAs such as those with 18 nucleotides. Adenine (A) also showed a moderate presence (Figure 3B). Graphs of miRNA nucleotide bias at each position for both the HF and LF groups showed distinct patterns in nucleotide representation across positions 1 through 22. At the first nucleotide position, uracil (U) was strongly predominant in both the HF and LF groups. As we moved from position 2 to 22, a mixture of all four nucleotides (G, C, U, and A) was observed, with uracil (U) and Adenine (A) generally seeming to have higher proportions than Guanine (G) and Cytosine (C) in many positions (Figure 3C,D). Novel miRNA first nucleotide bias is shown in Supplementary Figure S3.

#### 2.4.2. miRNA Expression

**Figure 4:**
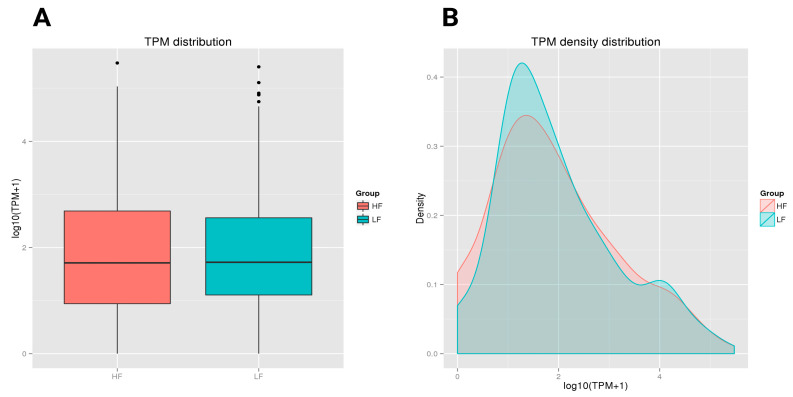
A boxplot of the log-transformed TPM expression and TPM density distribution of miRNAs in the HF and LF groups. The graphs compare the TPM distribution between the HF and LF groups. The boxplot shows similar median TPM values, with the HF group having slightly higher variability and more outliers. (**A**) A boxplot depicting the log-transformed TPM expression values of the HF and LF ram sperm samples. The solid horizontal line denotes the median, while the box contains the lower and upper quartiles in addition to the maximum and minimum values. TPM refers to Transcripts Per Million. (**B**) The TPM density distribution map elucidates the expression levels of transcripts in the HF and LF samples. The x-axis denotes the log-transformed TPM values, modified with a constant of 1 to prevent the log transformation of zero values. The y-axis represents density, denoting the ratio of transcripts at each expression level of miRNAs. The area under each curve was not normalized to 1; the curves represent relative differences rather than probability distributions.

Both the HF and LF groups showed a similar median TPM value with comparable central tendencies in expression levels. However, the HF group had a slightly wider interquartile range, with greater variability in expression levels among its samples compared to the LF group. Both groups also had a few outliers, with LF showing more outliers at the higher end of the expression range (Figure 4A). In the TPM density distribution plot, the HF and LF groups demonstrated different distribution patterns. The LF group, presented in green, had a slightly higher density peak around a log (TPM + 1) value of approximately 1.5. The HF group, shown in red, had a lower peak in this region. As the expression level increased (moving right on the x-axis), the density decreased for both groups. However, the tail on the right side showed that both the HF and LF groups contained some highly expressed transcripts, although these were less frequent (Figure 4B).

#### 2.4.3. Differentially Expressed miRNAs Between LF and HF

**Figure 5:**
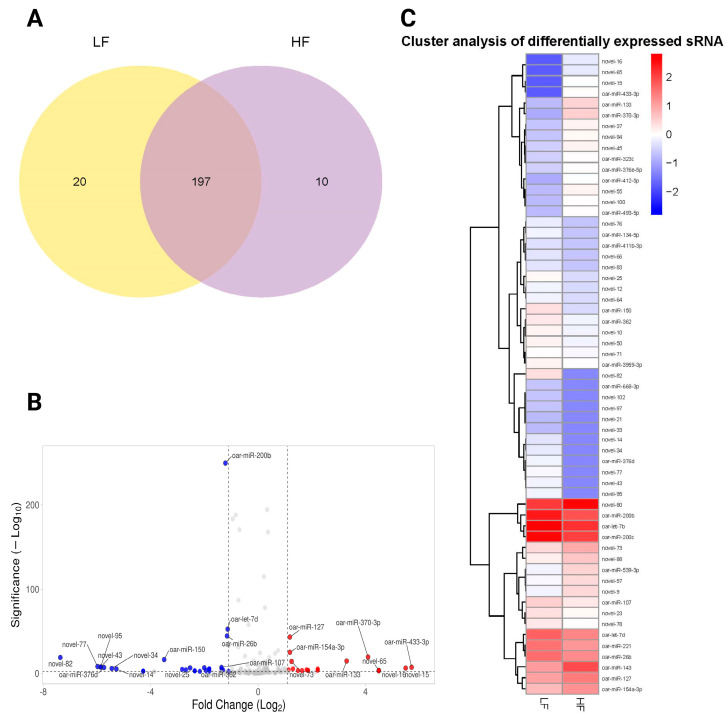
An analysis of differentially expressed miRNAs in the HF and LF groups. (**A**) The Venn diagram represents the number of miRNAs contained in each group (the LF label is aligned with the yellow circle, and HF is aligned with the purple circle), and the overlap represents the number of common miRNAs in the LF and HF groups. (**B**) The volcano plot shows differentially expressed miRNAs between the LF and GF rams (Red dots show upregulated miRNAs, blue dots show downregulated miRNAs, and gray dots represent non-significant miRNAs). (**C**) A heatmap of differentially expressed miRNAs between the LF and HF groups. Significantly upregulated and downregulated genes are colored in red and blue, respectively.

We illustrated the overlap and unique differentially expressed miRNAs between the LF and HF samples using the Venn diagram (Figure 5A). A total of 197 differentially expressed miRNAs were shared between the two groups, indicating a significant commonality in gene expression (Supplementary File S3). However, 20 differentially expressed miRNAs were unique to the LF group, while 10 differentially expressed miRNAs were specific to the HF group (Figure 5A). A volcano plot was generated to visualize the relationship between the significance and the fold-change of differentially expressed miRNAs. The plot highlighted several notable miRNAs with significant changes (FDR < 0.05), marked by their respective log2 fold-change and −log10 *p*-values. We found that 22 miRNAs were significantly upregulated, and 40 were downregulated between LF and HF groups. Key miRNAs, such as oar-miR-200b, oar-miR-26b, and oar-let-7d, were significantly downregulated, whereas others like oar-miR-127, oar-miR-370-3p, and oar-miR-154a-3p were upregulated. In addition, novel miRNAs such as novel_82, novel_95, and novel_77 were also differentially expressed (Figure 5B). Distinct clustering patterns were evident in the heatmap, clearly segregating miRNAs into groups associated with either LF or HF phenotypes. We showed that a subset of miRNAs, including oar-miR-200b, oar-miR-127, and oar-miR-370-3p, was upregulated in the HF group, forming a distinct cluster that separates from the LF-associated miRNAs. Conversely, miRNAs such as novel_77 and novel_43 demonstrated downregulation in the LF group (Figure 5C).

### 2.5. Functional Interaction Network Association Analysis of Target Genes in LF and HF Groups

**Figure 6:**
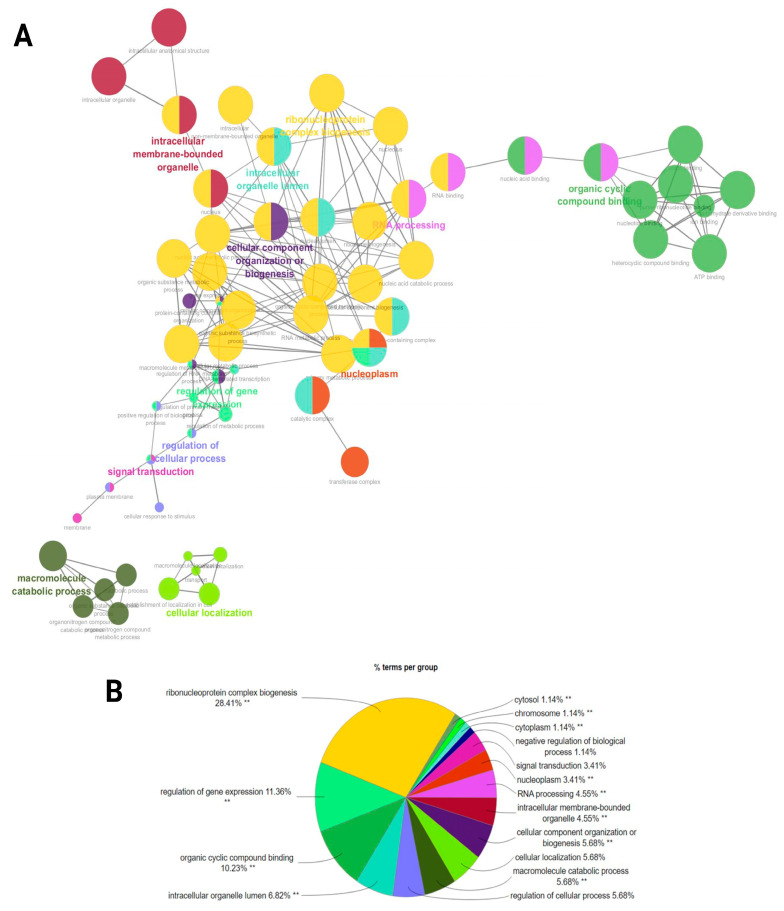
A functional network association analysis of the target genes from ram sperm. (**A**) The GO terms are shown as nodes, and the color depths of the nodes show distinct proportions of target genes. Nodes located in the same cluster have the same node color, and the sizes of nodes demonstrate the number of mapped genes in each GO term. (**B**) An overview chart indicating functional target genes in the sperm of HF and LF rams. Double (**) asterisks indicate significantly enriched GO terms at the *p* < 0.01 statistical level.

A functional network association analysis of miRNA target genes revealed critical insights into their regulatory roles across various biological processes and molecular functions as well as cellular components (Figure 6A). The network visualization demonstrated key hubs such as (GO:0022613) ribonucleoprotein complex biogenesis, (GO:0010468) the regulation of gene expression, and (GO:0006396) RNA processing. Highly interconnected clusters demonstrated the central roles of (GO:0071840) cellular component organization or (GO:0043231) intracellular membrane-bounded organelle processes (Figure 6A). The bar chart underscores the distribution of miRNA targets across enriched biological processes. Among these, (GO:0022613) ribonucleoprotein complex biogenesis stands out as the most enriched term, further supported by categories such as (GO:0003723) RNA binding, (GO:0007165) signal transduction, and (GO:0071840) cellular component organization or biogenesis, indicating the miRNA-mediated regulation of cellular communication and structural organization. The pie chart provides a quantitative breakdown, with ribonucleoprotein complex biogenesis representing the largest proportion (28.41%) of functional terms. This is followed by the regulation of gene expression (11.36%) and organic cyclic compound binding (10.23%), confirming the pivotal role of miRNAs in transcriptional regulation and ligand binding. Smaller categories include signal transduction (3.41%) and the negative regulation of biological processes (1.14%) (Figure 6B) (Supplementary File S4).

## 3. Discussion

In the selection of superior males in livestock as a source of frozen semen, cellular analyses alone are no longer considered reliable predictors of male fertility [2,21], Hitit and Memili, 2022. Applications of methods for analyzing the sperm transcriptome, such as monitoring RNA levels, are expected to increase fertility assessments of sperm [22]. The widespread use of this technology in livestock production has established sperm transcriptome analysis as a primary tool for predicting male fertility potential in the farm industry [19,23]. Along this line, our study demonstrated the sncRNA profiles in sperm from rams with HF and LF phenotypes in order to identify prospective sncRNAs that are linked to fertility.

Through comprehensive sncRNA profiling of ram sperm, we identified 14,962,876 reads in the LF sample with a total base count of 0.75G, while the HF sample produced 17,401,094 reads with 0.87G bases. Likewise, Ureña et al. [24] successfully aligned 16.7 million ovine ejaculate read pairs to the sheep genome. The length distribution of sncRNAs in sperm typically reached the highest length at around 28–32 nucleotides, with the majority of sperm sncRNAs varying within the range of 18–35 nucleotides, reflecting the characteristic size of small non-coding RNAs found in sperm cells; however, the exact distribution can vary depending on the species: in bull sperm, the majority of small non-coding sncRNAs seems to vary within the 18–30 nucleotide length range, with a significant portion of reads being specifically around 27–32 nucleotides [25]. On the contrary, the length distribution of sncRNAs demonstrated that the major reads from pig sperm were 18–26 nt [26]. In both the HF and LF samples, the miRNAs demonstrated a dominant bias to U at the first nucleotide, especially miRNAs with lengths of 18–30 nt, except for the length of 25 nt, which was biased toward A. Also, species such as mouse, human, and rat spermatozoa had strong bias towards U [20], and similarly, in line with our study, bovine spermatozoa had the same U bias trend between and HF and LF phenotypes [27]. Our results show that sperm cells had U bias, which agrees with the characteristic that miRNAs often begin with a U at the 5′ terminus [28].

Sperm contains a diverse array of sncRNAs of which biotypes were identified in the sperm of species investigated in livestock, including bovine, ovine, and pig [24,29,30]. These sncRNAs comprised mostly rRNAs, tRNAs, tsRNAs, snoRNAs in bull sperm [29] and were shown to be involved in spermatogenesis and in determining male fertility. Consistent with previous reports, we demonstrated diverse sncRNA populations in ram sperm. For instance, the most abundant classes of sncRNA in ram sperm were rRNA, snoRNA, snRNA, and tRNA. Similarly, in sheep sperm, rsRNA, miRNA, and tsRNA were the most abundant sncRNA biotypes [26]. In boar sperm, Gòdia et al. [30] also showed that 34% of reads were aligned to sncRNAs, most of which were miRNAs and tRNAs. Although the repertoire of RNA populations in sperm is diverse, their functional role has remained unknown in the literature as well as in ovine spermatozoa.

It was intriguing that altered miRNA expression may be a potential biomarker to determine fertility and predict sperm functions in bulls [31,32]. In this study, we identified 1673 known and 627 novel miRNAs in ram sperm, showing distinct patterns of differential expression between the HF and LF phenotypes. Of these, 227 differentially expressed sncRNAs were linked with sperm from HF and LF rams. In comparison to our study, bull sperm possessed 1431 miRNAs, of which there were only five sequences that were shown to be differentially expressed between fertility groups [33], while Keles et al. [34] identified 85 differentially expressed miRNAs in cryopreserved bull semen linked to fertility. The distinct clustering of miRNAs observed in the HF and LF groups suggests that specific regulatory networks underlie fertility phenotypes. For instance, miRNAs such as oar-miR-200b and oar-miR-370-3p were significantly upregulated in the HF group; likewise, bovine mir-200b is highly expressed in HF bull spermatozoa and is also expressed in sperm during their transit through the epididymis [35]. Similarly, oar-miR-370-3p was shown to be involved in epididymal sperm maturation [36] and motility [37]. Additionally, miR-200b targets specific genes associated with spermatogenesis [38]. This targeting mechanism may underscore the regulatory role of ovine miRNAs in sperm development and function. Conversely, the downregulation of miRNAs like oar-miR-26b and oar-let-7d in the LF group may point to disruptions in cellular processes, which altered the expression of miR-26b, and let-7d was shown to be associated with spermatogenic impairments [39], morphological abnormalities [40], and an elevated sperm DNA fragmentation index [41], which may explain the reduced fertility in LF rams.

A functional network analysis of miRNA target genes provided further evidence of their regulatory significance. The enrichment of GO terms such as (GO:0022613) ribonucleoprotein complex biogenesis, (GO:0010468) the regulation of gene expression, and (GO:0006396) RNA processing suggests that miRNAs exert a broad influence over essential biological processes that may govern sperm functionality. As such, spermatogenesis requires precise spatial and temporal regulation of gene expression, where miRNAs are expressed in a cell-specific or stage-specific manner during spermatogenesis [16,42]. We showed that *CDK4*, *DROSHA*, *EEF2*, *HDAC3*, *NCBP1*, *HSPA8*, and *TRAF6* were commonly enriched in the network of gene expression. Of these, *DROSHA*, a target gene, is an RNase III enzyme and a cofactor of DGCR8 in the nucleus that mediates the processing of miRNA primary transcript in spermatozoa [43], and impaired expression caused deficiency in miRNA synthesis and infertility [17]. Eukaryotic elongation factor 2 (*EEF2*) is another potential target gene that might be crucial for protein synthesis during spermatogenesis because studies indicated that disruptions in EEF2 function can lead to male infertility, as seen in transgenic mice models with premature mRNA translation [44]. Moreover, miRNAs have shown to be involved in chromatin remodeling; Bedi et al. [45] noted that maturing sperm contains HDAC3, suggesting its involvement in chromatin alterations that refine the paternally inherited epigenome. Further explorations of GO terms in relation to miRNA target genes revealed that sperm ribonucleoprotein complexes are one of the key networks that modulate development, maturation, and fertilization; this aligns with and extends previous findings on mechanistic trials of sperm-borne miRNAs potentially regulate gene expression during early embryonic development [17,46].

Our future research will focus on validating key differentially expressed miRNAs, particularly oar-miR-200b, oar-miR-370-3p, oar-miR-26b, and oar-let-7d, due to their strong association with ram sperm function and fertility. Additionally, further functional analyses, including target gene validation and gene expression studies, will be conducted to explore the role of novel miRNAs in spermatogenesis and early embryonic development.

## 4. Materials and Methods

### 4.1. Ram Fertility Assessment and Experimental Design

Data on fertility phenotypes of adult rams (Anatolian Merino) were collected at the Republic of Türkiye’s Ministry of Agriculture and Forestry’s Institute of Bahri-Dadaş International Agricultural Research. We used pregnancy rates from natural mating to predict the fertility of mature rams (*n* = 66) that were at least 3–4 years old throughout the breeding seasons of 2017–2019. Using teaser rams, the ewes’ estrus was detected; these rams were prohibited from mating by covering the prepuce region. The ewes were introduced to the teaser males in the early morning for approximately thirty minutes. Estrus was regarded as the ewes’ attempt at teasing and their acceptance of teaser ram mount attempts. For natural mating, a handler introduced estrous females into an enclosure with a single ram selected at random. Estrus detection was maintained throughout the breeding season, and ewes were permitted to mate with the selected male at random. Ewes were considered to be pregnant after 35 days of mating provided that they failed to come to estrus. Furthermore, the quantities of pregnant and non-pregnant ewes for each ram were determined by assessing the dates of mating and lambing with the duration of the pregnancies. The conception rates of the ewes were utilized to calculate their fertility scores. We grouped the rams into two phenotypes based on their fertility levels: the HF group (*n* = 31; 94.5 ± 2.8%) and the LF group (*n* = 25; 83.1 ± 5.73%). We calculated the average pregnancy rate as 89.4 ± 7.2% (*n* = 66), and the rams were grouped into these groups based on their fertility levels. Each ram was enrolled to serve at least 50 ewes in both groups in the breeding season.

### 4.2. Semen Collection

Animal procedures were approved by Bahri-Dagdaş Research Center Ethical Committee, Türkiye (Number: 22.12.2016/58). Rams were trained to ejaculate semen using an artificial vagina (AV) that allow them to mount on teaser ewes during heat. Rams were allowed to ejaculate into the AV upon mounting. We discarded the first three collections prior to the collection of research samples, followed by semen collection and processing for use in research. The fertility rates that were considered to be outliers were those that were either one standard deviation above or below the mean. We selected four rams with the highest fertility (pregnancy rate; % 99.2 ±1.6) and four rams with the lowest fertility (pregnancy rate; % 73.6 ±4.4) for the purpose of sequencing sncRNA with a high level of confidence. Following that, we collected approximately 2 × 10^9^/mL of spermatozoa from each ejaculate. Then, the aliquots from each sample were adjusted to a final concentration of 10^7^/mL inside of straws, and then they were frozen at −80 °C until the sncRNA analysis was performed [5,19].

### 4.3. RNA Isolation

The sperm samples were filtered through a sieve with a mesh size of 500 in order to remove any cell debris that were present before the RNA was extracted. Then, the samples of sperm were passed through a somatic cell lysis solution consisting of 0.3% Triton X-100 and 0.1% SDS in DEPC-treated water for a total of twenty-five minutes on ice and washed two times. After washing, sperm integrity was confirmed under a microscope, and the absence of somatic cell contamination was verified by Real-Time PCR analysis targeting PRM1 and PRM2 genes. This was carried out in order to eliminate somatic cells, and then the examination of non-sperm cell contamination was carried out using a microscope. Using the SanPrep column microRNA miniprep kit (Bio Basic Inc., Markham, ON, Canada) with little modification and following the instructions provided by the manufacturer, total RNA was extracted from the purified ram sperm samples (*n* = 4, for each group). On the pellet, we introduced 800 microliters of a guanidine thiocyanate lysis buffer that was enriched in 20 millimolar DL-Dithiothreitol. Subsequently, we homogenized the sperm cells by running the samples through a 26-gauge needle syringe twenty to twenty-five individual times. Following the complete elimination of any additional contaminants and the attachment of total RNA to the membrane, on-column DNase digestion was carried out in order to eliminate any residues of DNA contamination. In order to determine the concentration and integrity of the total RNA samples, we utilized a NanoDrop (Colibri Microvolume Spectrometer, Titertek-Berthold, Pforzheim, Germany) and a 2100-Bioanalyzer along with the RNA 2100 Nano Chip (Applied Biosystems, Carlsbad, CA, USA), respectively.

### 4.4. Library Preparation for sncRNA Sequencing

For small RNA sequencing library construction, 1 μg of total RNA per sample was utilized. Then, using the TrueSeq Small RNA Library Prep Kit (Illumina, San Diego, CA, USA) in compliance with the commercial kit protocol, sequencing libraries were developed. We, briefly, purified the total using polyacrylamide gel electrophoresis (PAGE) to obtain small RNAs that were 18–40 nucleotides (nt) in length. Consequently, we ligated the NEB 3′ SR adaptor to the 3′ end of the small RNA fragments; the double-stranded DNA adaptor was transformed; 5′ end adapters were ligated to the 5′ ends of the small RNA fragments; and cDNA was generated by a reverse transcription reaction. Following purification and size selection, we checked the library with Qubit and a bioanalyzer (Agilent Technologies, Santa Clara, CA, USA) for size distribution detection. We pooled quantified libraries and carried out sequencing using the HiSeq 2500 system (Illumina, San Diego, CA, USA) with a read length of 50 bp.

### 4.5. Alignment of RNA-Seq Reads and Assembly of Transcripts

We initially filtered the raw reads of small RNA sequencing to obtain clean data for later analysis through the following steps: We discarded low quality reads, reads containing poly-N, reads shorter than 18 nt, reads with 5′ adapter contaminants, and reads without a 3′ adapter insert and tag. Then, all aligned clean reads were aligned to the *Ovis aries* (v4.0) reference genome using Bowtie v2.0.6 [47]. We confirmed trimmed reads to ensure the quality threshold (Q-score; Q20 and Q30) with no bias in the evaluation. Quality scores were determined using the Phred scale, which measures the reliability of base calls. Q20 denotes a base call accuracy of 99% (or 1% risk of error), whereas Q30 denotes a base call accuracy of 99.9% (or 0.1% chance of error). To search for known miRNA, mapped small RNA tags were used and then aligned to the miRBase20.0 database (http://www.mirbase.org/; accessed on 15 February 2025). In addition, through the unaligned sequences, potential novel miRNA and secondary structures were identified using the miREvo_v1.1 and mirdeep2_0_0_5 software because of the hairpin structure in pre-miRNA [48,49]. Moreover, small RNA reads, including rRNAs, tRNAs, snRNAs, snoRNAs, repeat sequences, exons, and introns, were also identified, respectively.

### 4.6. miRNA and Analysis of Differentially Expressed microRNAs and Target Ene Prediction

The miRNA expression levels of both known and novel miRNAs were quantified by Transcripts Per Million (TPM), where it was normalized as the mapped read count/total reads × 1,000,000 libsize. We identified differentially expressed microRNAs (DEMs) using the R package DESeq 1.8.3. Differential expression analysis between the HF and LF samples was performed using the DEGseq (2010) R package. The miRNAs showing *p* < 0.05 and |log2-fold| change ≥ 1 in comparison were set as the threshold for significantly differentially expressed miRNAs (DEmiRNAs) [50,51]. Target gene prediction of differentially expressed miRNAs from sperm samples from HL and LF rams was performed using the miRNAconsTarget online tool from sRNAtoolbox (https://arn.ugr.es/srnatoolbox/amirconstarget/; accessed on 5 February 2025), generating consensus target prediction. The uploaded input data were predicted based on independent prediction from animal-based tools. They were TargetSpy, PITA (energy score < −15), and miRanda (pairing score >150 and an energy score < −15), providing a total of three prediction algorithms. We included the common target genes estimated by all three tools as potential miRNA targets.

### 4.7. Protein–Protein Interaction Network and Gene Ontology and Pathway Enrichment Analysis of Target Genes

We established the functional network association among target genes using the database of STRING (version 12.0, http://string-db.org; accessed on 4 February 2025), and eventually visualized it in Cytoscape (version 3.10.1). The PPI network of target genes was transferred and subsequently assessed in Cytoscape. We predicted the KEGG pathway and GO enrichment for the predicted target genes from the network analyzed using Cytoscape software with the ClueGO V2.5.10 plug-in [52]. The ClueGO plug-in produced functionally grouped GO annotation networks for target genes. We accepted GO terms possessing a corrected *p*-value < 0.05 as being significantly enriched by DE genes. We assigned the GO categories as molecular function (MF), cellular component (CC), and biological process (BP). We used two-sided hypergeometric tests with the *p*-value of 0.05, and multiple test corrections were performed using Bonferroni step-down adjustment. The threshold of the kappa score was adjusted to 0.5.

### 4.8. Statistical Analysis

In order to analyze the data, the SPSS software (version 22.0) was utilized. The software GraphPad Prism 9 (GraphPad Software, San Diego, CA, USA) was utilized in order to construct statistical graphs. During the course of the experiment, there were four biological replicates involved, and each measurement was carried out twice. An independent *t*-test was used to examine the differences between the LF and HF groups. The significance level was determined to be 0.05.

## 5. Conclusions

The current study provides novel insights into the sncRNA profiles of ram sperm and their association with fertility. We identified key miRNAs, such as oar-miR-200b and oar-miR-370-3p, that were upregulated in HF rams, while miRNAs like oar-miR-26b and oar-let-7d were downregulated in LF rams. Similar patterns have been observed in bovine studies [35], where miR-200b and miR-370-3p play roles in sperm function and fertility. The differential expression of miRNAs and their enrichment in key biological pathways highlighted their regulatory importance in sperm functionality and control of sperm fertility. Our findings have significant implications for livestock breeding, offering a molecular basis for the development of biomarkers to assess ram fertility. Integrating these markers can possibly enhance reproductive efficiency and productivity in sheep farming, contributing to global food security.

## Figures and Tables

**Table 1:** Summary of data production.

Sample	Reads	Bases	Error Rate	Q20	Q30	GC Content
LF	14,962,876	0.75G	0.01%	98.98%	96.62%	53.11%
HF	17,401,094	0.87G	0.01%	98.52%	95.45%	52.99%

Sample ID (low and high fertility, LF and HF); reads: statistics of original sequence data, bases: sequence number multiplied by length of sequence, expressed in gigabase pairs (Gbp); error rate: sequencing error rate; Q20: percentage of bases whose Phred values exceed 20; Q30: percentage of bases whose Phred values exceed 30; GC content: G and C bases as percentage of all bases.

**Table 2:** Types and quantity of sRNA.

Sample	Total Reads	Total Bases (bp)	Unique Reads	Unique Bases (bp)
LF	9,851,512	269,292,004	1,550,984	40,934,899
HF	10,611,165	303,347,912	1,494,593	41,404,805

Sample ID (low and high fertility, LF and HF); total reads: total number of sRNA reads; total bases (bp): total reads multiplied by sequence length; unique reads: types of sRNA; unique bases (bp): unique reads multiplied by sequence length.

## Data Availability

The datasets analyzed are available in the EMBL-EBI HYPERLINK at https://www.ebi.ac.uk/biostudies/studies/S-BSST1872 (accessed on 5 February 2025) accessible at the following accession number: “S-BSST1872”. The repository contains all raw relevant data.

## Supplementary Materials

The following supporting information can be downloaded at https://www.mdpi.com/article/10.3390/ijms26062690/s1.
