# Supplementary material for: Exploration of Small Non-Coding RNAs as Molecular Markers of Ram Sperm Fertility
Source: Int J Mol Sci. 2025 Mar 17;26(6):2690. doi: 10.3390/ijms26062690 (PMC11942391; doi:10.3390/ijms26062690)

Supplementary Figure S1. Comparison of unique and total small non-coding RNAs (sncRNAs) between LF and HF conditions.

Figure Legend S1: (A) Venn diagram representing the distribution of unique sncRNAs between Low Fertility (LF) and High Fertility (HF). The red circle indicates LF specific sncRNAs (45.25%), the blue circle represents HF specific sncRNAs (43.18%), and the overlapping region (purple) denotes sncRNAs common to both conditions (11.57%). (B) Venn diagram depicting the distribution of total sncRNAs between LF and HF. LF specific sncRNAs account for 9.52% (red), HF specific sncRNAs make up 9.27% (blue), while 81.2% of the total sncRNAs are shared between the two conditions (purple).

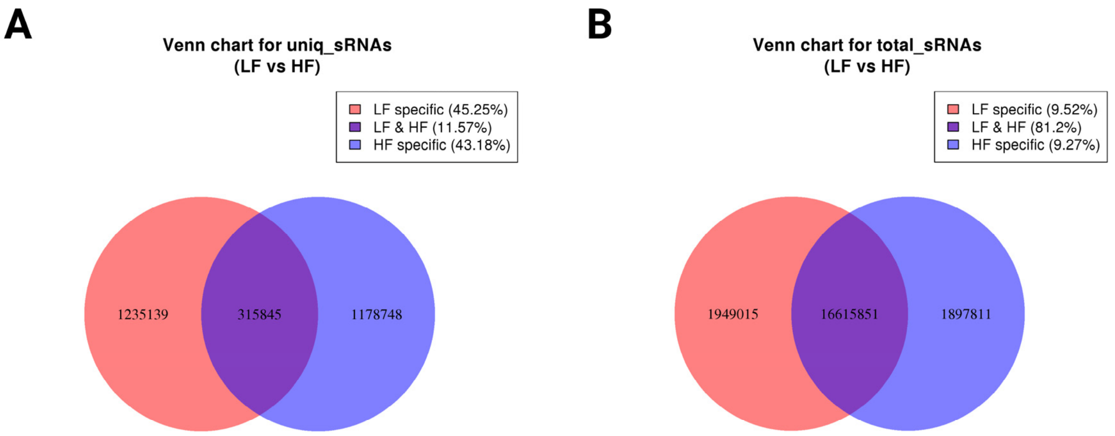

Supplementary Figure S2. The distribution of total reads of non-coding RNA biotypes reads in HF and LF conditions.

Figure Legend S2: (A) Pie chart depicting the annotation of unique sncRNA reads in the HF condition. The distribution includes various RNA categories, such as exon-derived, intron-derived, known and novel miRNAs, repeat elements, ribosomal RNAs (rRNA), small nucleolar RNAs (snoRNA), small nuclear RNAs (snRNA), and transfer RNAs (tRNA). The "other" category represents a substantial proportion of the unique reads. (B) Pie chart illustrating the annotation of unique sncRNA reads in the LF condition, showing a similar distribution to the HF condition, with variations in the proportion of different RNA classes.

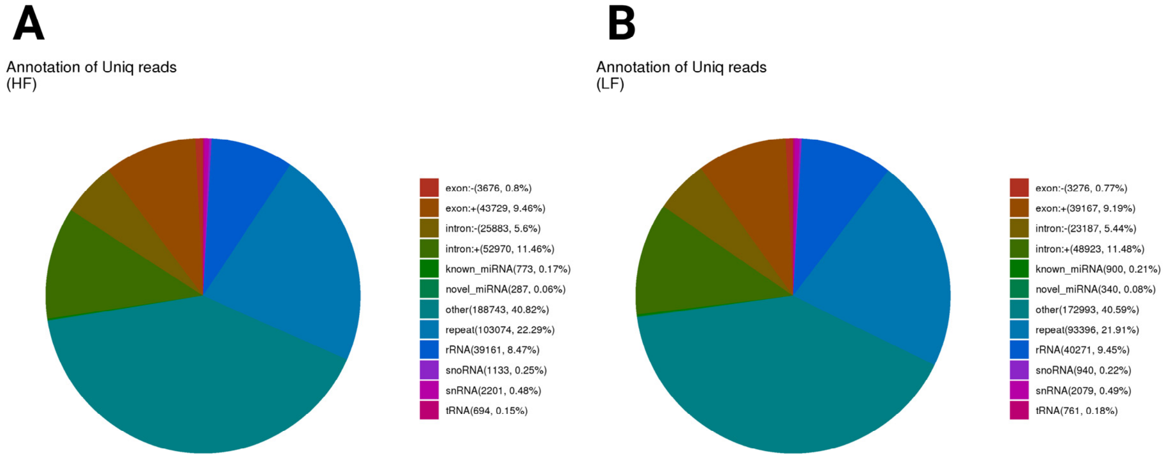

Supplementary Figure S3. First nucleotide bias of unique miRNAs in HF and LF conditions across different sequence lengths.

Figure Legend S3: (A) Distribution of the first nucleotide bias of miRNAs in the HF condition across miRNA lengths ranging from 18 to 30 nucleotides (nt). The bar plots represent the proportion of each nucleotide (G, C, U, A) at the first position, with colors corresponding to different nucleotides. The numbers above each bar indicate the count of miRNAs of a given length. (B) First nucleotide bias of miRNAs in the LF condition, following the same structure as (A). The distribution highlights differences in the preference for U, A, G, and C at the first position across miRNA lengths.

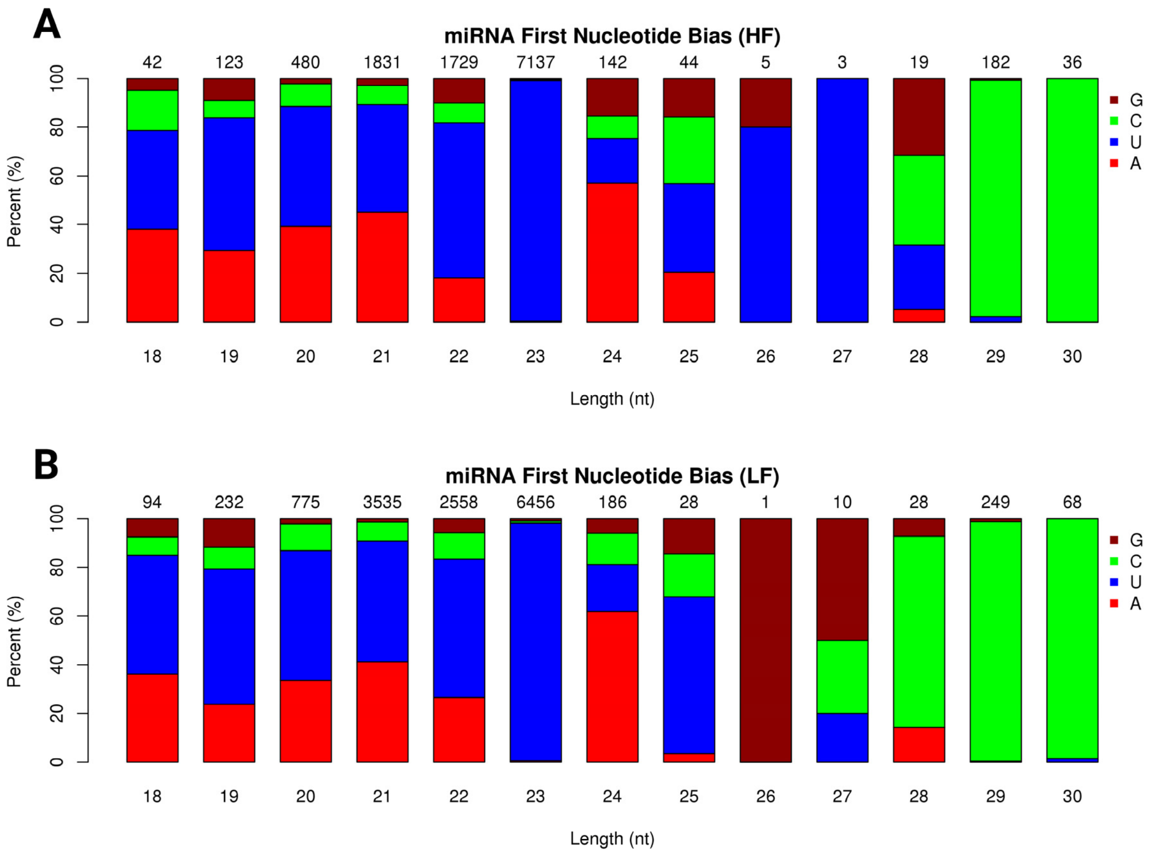

Supplement: Supplementary file 1 [file ijms-26-02690-s001.zip › figure captions and legends.pdf]
